# Acceptability of a Hypothetical Reduction in Routinely Scheduled Clinic Visits Among Patients With History of a Localized Melanoma (MEL-SELF): Pilot Randomized Clinical Trial

**DOI:** 10.2196/45865

**Published:** 2023-06-26

**Authors:** Dorothy Drabarek, Deonna Ackermann, Ellie Medcalf, Katy J L Bell

**Affiliations:** 1 Sydney School of Public Health Faculty of Medicine and Health The University of Sydney Sydney Australia

**Keywords:** melanoma, surveillance, medical overuse, teledermatology, pilot, clinical visit, treatment, clinics, monitoring, self-examination, online questionnaire, diagnosis, patient, safe, effective

## Abstract

**Background:**

After treatment for a localized melanoma, patients attend routinely scheduled clinics to monitor for new primary or recurrent melanoma. Patient-led surveillance (skin self-examination with patient-performed teledermoscopy) is an alternative model of follow-up that could replace some routinely scheduled visits.

**Objective:**

This study aims to assess the acceptability of a hypothetical reduction in routinely scheduled visits among participants of the Melanoma Self Surveillance (MEL-SELF) pilot randomized clinical trial of patient-led surveillance (intervention) versus usual care (control).

**Methods:**

Patients previously treated for localized melanoma in New South Wales who were participating in the MEL-SELF pilot randomized clinical trial were asked to respond to a web-based questionnaire at baseline and after 6 months on trial. We used mixed methods to analyze the data. The main outcome of interest was the acceptability of a hypothetical reduction in routinely scheduled visits for melanoma surveillance.

**Results:**

Of 100 randomized participants, 87 answered the questionnaire at baseline, 66 answered the questionnaire at 6 months, and 79 provided a free-text explanation at either time point. At 6 months, 33% (17/51) of the control group and 35% (17/49) of the intervention group indicated that a hypothetical reduction in routinely scheduled visits with all melanoma doctors was at least slightly acceptable (difference in proportions –1%, 95% CI –20% to 17%; *P*=.89). Participants suggested that prerequisites for a reduction in routinely scheduled visits would include that sufficient time had elapsed since the previous diagnosis without a new primary melanoma or recurrence, an unscheduled appointment could be made at short notice if the patient noticed something concerning, their melanoma doctor had suggested reducing their clinic visit frequency, and patients had confidence that patient-led surveillance was a safe and effective alternative. Participants suggested that a reduction in routinely scheduled visits would not be acceptable where they perceived a very high risk of new or recurrent melanoma, low self-efficacy in skin self-examination and in the use of technologies for the patient-led surveillance intervention, and where they had a preference for clinician-led surveillance. Some patients said that a partial reduction to once a year may be acceptable.

**Conclusions:**

Some patients may be receptive to a reduction in routinely scheduled visits if they are assured that patient-led surveillance is safe and effective.

**Trial Registration:**

Australian New Zealand Clinical Trials Registry ACTRN12616001716459; https://www.anzctr.org.au/Trial/Registration/TrialReview.aspx?id=371865&isReview=true; ClinicalTrials.gov NCT03581188; https://clinicaltrials.gov/ct2/show/NCT03581188

**International Registered Report Identifier (IRRID):**

RR2-10.1001/jamadermatol.2021.4704

## Introduction

Patient-led surveillance has the potential to replace some routinely scheduled clinic visits after treatment of primary localized melanoma [1-3], offering a more sustainable model of follow-up care [4]. We recently completed a pilot randomized clinical trial (RCT) to assess the safety, feasibility, and acceptability of patient-led surveillance compared to clinician-led surveillance [5]. As part of the pilot trial, we asked participants about the acceptability of a hypothetical reduction in routinely scheduled clinics.

## Methods

### Overview

The Melanoma Self Surveillance (MEL-SELF) pilot RCT included patients previously treated for localized melanoma (stages 0, 1, and 2) who owned a smartphone, had a skin check partner to assist with skin self-examination (SSE), and were attending a routinely scheduled follow-up. Participants were recruited from specialist and primary care clinics in New South Wales, Australia. Participants were randomized (1:1) to 6 months of patient-led surveillance (intervention: reminders to perform SSE, patient-performed dermoscopy, teledermatologist assessment, and fast-tracked unscheduled clinic visits, in addition to usual care) or usual care (control). The pilot trial protocol is provided in Multimedia Appendix 1.

Prior to randomization, potential participants were provided with a participant information statement that included information about the patient-led surveillance intervention (Textbox 1). Participants assigned to the control group did not experience any components of the patient-led surveillance intervention.

In a web-based survey at baseline and at 6 months (delivered via REDCap [Research Electronic Data Capture]; Vanderbilt University [6,7]), participants were asked questions about the acceptability of a hypothetical reduction in routinely scheduled clinic visits at baseline and 6 months (Multimedia Appendix 2 [6,7]). For the quantitative analysis, we undertook an intention-to-treat analysis of outcomes at 6 months. We undertook an exploratory subgroup analysis by American Joint Committee on Cancer substage (AJCC; melanoma in situ, AJCC 0 compared with invasive melanoma, or AJCC 1-4). We used standard formulas to estimate the *P* values. We undertook statistical analysis using RStudio 2022.2.2.485 (RStudio, PBC). For the qualitative analysis of free-text answers, we used content analysis of free-text explanations to group them into themes. Two authors inductively developed codes and themes from the data.

**Text from participant information statement provided to all participants about the patient-led surveillance intervention.**

“Most melanomas are detected by patients or their family members between scheduled visits; even more might be detected if patients are trained in total body skin self-examination (SSE). The objective of this study is to investigate whether a Smartphone App with videos showing how to perform skin self-examination and teledermoscopy (taking close up photographs of your skin using your phone) may lead to performing skin self-examination more regularly and increases confidence in doing this compared to standard education to learn about skin self-examination from a booklet alone (usual care).”

“If you are in the ‘intervention’ group you will also receive:

- A dermatoscope (this device allows you to take magnified images of skin lesions under polarized light for electronic transmission to a specialist) to attach to a smartphone and work in conjunction with a Smartphone App. A dermatologists will review the reports and images you submit in the app within 3 working days (if not the study coordinator will notify you with information on the delay). The dermatologist will then provide you with a clinical recommendation after they review your images.

- Written and video instructions on how to use the dermatoscope and the associated Smartphone App.

- Email and SMS text reminders every 2 months to perform self-examination on the Smartphone App and to complete a survey. The skin checker survey will also provide you with instructional videos on guided total skin self-examination and electronic reporting.”

### Ethics Approval

This study was approved by the Sydney Local Health District Ethics Committee (HREC/15/RPAH/593). All participants provided informed consent. The reporting of this study followed the CONSORT-EHEALTH (Consolidated Standards of Reporting Trials of Electronic and Mobile Health Applications and Online Telehealth) guidelines [8].

## Results

### Overview

This study was conducted from November 1, 2018, to January 17, 2020. Of the 100 trial participants, 87 answered questions at baseline, and 66 answered at 6 months (Table 1 and Multimedia Appendix 3). In addition, 71 participants provided a free-text explanation for their answers at baseline, and 56 provided this at 6 months.

**Table 1:** Effects of the intervention on the acceptability of a hypothetical decrease in routinely scheduled visits at the 6-month follow-up.^a,b^

			Control (n=51), n (%)				Intervention (n=49), n (%)				Total (N=100), n (%)		
			Baseline		Follow-up		Baseline		Follow-up		Baseline		Follow-up
**Acceptability of a decrease in scheduled visits with all melanoma doctors (specialists and GP^c^)**													
	Not acceptable	21 (41)		19 (37)		23 (47)		13 (27)		44 (44)		32 (32)	
	Slightly/somewhat acceptable	19 (37)		14 (27)		16 (33)		15 (31)		35 (35)		29 (29)	
	Very/completely acceptable	4 (8)		3 (6)		4 (8)		2 (4)		8 (8)		5 (5)	
**Acceptability of a decrease in scheduled visits with GP**													
	Not acceptable	13 (25)		9 (18)		11 (22)		9 (18)		24 (24)		18 (18)	
	Slightly/somewhat acceptable	18 (35)		17 (33)		20 (41)		17 (35)		38 (38)		34 (34)	
	Very/completely acceptable	13 (25)		10 (20)		12 (24)		4 (8)		25 (25)		14 (14)	
**Acceptability of a decrease in scheduled visits with melanoma specialist**													
	Not acceptable	22 (43)		18 (35)		21 (43)		15 (31)		43 (43)		33 (33)	
	Slightly/somewhat acceptable	16 (31)		14 (27)		18 (37)		13 (27)		34 (34)		27 (27)	
	Very/completely acceptable	6 (12)		4 (8)		4 (8)		2 (4)		10 (10)		6 (6)	

^a^Percentages may not sum to 100 owing to rounding.

^b^Missing data at baseline for 7 (14%) participants in the control group and 6 (12%) participants in the intervention group and at follow-up for 15 (29%) in the control group and 19 (39%) in the intervention group.

^c^GP: general practitioner.

### Quantitative Analysis

We measured the acceptability of a hypothetical reduction in routinely scheduled visits with all melanoma doctors on a 5-point Likert scale. After dichotomizing this outcome (not acceptable vs slightly/some/very/completely acceptable), there was no difference between the randomized groups in the acceptability of reducing routinely scheduled visits at 6 months, with 33% (17/51) of participants in the control group and 35% (17/49) of participants in the intervention group indicating that a hypothetical reduction was at least slightly acceptable (difference in proportions –1%, 95% CI –20% to 17%; *P*=.89).

Among all 100 participants, 34% (95% CI 25%-44%; *P=*.002) indicated that a reduction in routinely scheduled visits was at least slightly acceptable, with 5% (95% CI 2%-11%; *P*<.001) indicating it was very or completely acceptable and 29% (95% CI 20%-39%; *P*<.001) indicating it was slightly or somewhat acceptable. On the other hand, 32% (95% CI 23%-42%; *P*<.001) indicated that a reduction in routinely scheduled visits overall would not be acceptable (there was no response from 34%, [34/100] of participants for this question at 6 months).

These proportions were also similar for patients with melanoma in situ compared to patients with invasive melanoma in the exploratory subgroup analysis. At 6 months, 31% (11/36) of participants with melanoma in situ and 36% (23/64) with stage 1 or 2 melanoma indicated that a hypothetical reduction in scheduled visits was at least slightly acceptable (difference in proportions –5%, 95% CI –24% to 14%; *P=*.59). More detailed results on this exploratory subgroup analysis are presented in Multimedia Appendix 4.

### Qualitative Analysis

The free-text explanation responses were similar across the randomized arms and indicated a number of broad themes as to when a reduction would (Textbox 2) and would not be acceptable (Textbox 3).

**Circumstances in which a reduction in routinely scheduled visits would be acceptable (note, general practitioner [GP] in Australia is equivalent to primary care physician in North America).**

**Sufficient time had passed without any subsequent new melanomas**

- “I would potentially be more comfortable monitoring it without their supervision as I get further from my surgery without any repeats.” (015_control_6months)
- “If there has been no further problems or issues l can see no reason why they cannot be reduced.” (034_control_6months)
- “Maybe go to yearly check with specialist if nothing found on next visit which is this Month/ October.” (095_control_6months)
- “Acceptable as I am presently on 6 monthly checks that will be completing in March 2020 as it will be 5yrs since having my lesion removed. Will continue own skin examinations and visit GP if there is anything abnormal.” (017_intervention_6months)

**Timely access to a specialist facilitated by teledermatology and fast-tracked clinic visits**

- “If there are any problems or issues develop prior to you next scheduled visit, l can make an appointment see my GP or specialist.” (034_control_6months)
- “but if anything worries me I photo and email or book a short notice check.” (064_control_6months)
- “It would be acceptable provided if any issues the moles etc could be emailed, photos sent etc for confirmation they are ok.” (061_control_baseline)
- “I feel fewer scheduled visits would be ok if you are reassured it is ok to call with concerns and you can be guaranteed to be seen within a short time frame (1-2 weeks).” (072_control_6months)
- “I think knowing that I have access to them with concerns (not waiting too long for an appointment) I would feel ok with this.” (072_contol_baseline)

**Confidence in alternative to scheduled visits with or without digital technology**

- “In the longer-term future if I was confident there were other ways of checking, I'd be very happy to go less. But don't have confidence in my GP (they just send me to Melanoma specialist) or in my own detection skills.” (066_control_baseline)
- “Fewer follow up visits introduces an amount of uncertainty - unless good instructions are provided about using an I-phone to discover any problem areas.” (085_intervention_baseline)
- “Only if given tools/devices that I would be confident in self-examination.” (100_control_baseline)
- “I'm more confident in checking self.” (059_control_6months)
- “If I had confidence in technology reducing risk.” (089_control_baseline)
- “Provided my examinations were satisfactory in early detection it would remove anxiety and thus less unnecessary visits.” (064_control_baseline)
- “I like the idea of a specialist yearly check, but very happy to do in between checks by myself with added technology to help. But if technology is good, maybe no scheduled checks, then.” (070_control_baseline)
- “With the digital technology I am a little more confident of picking up a melanoma early.” (011_intervention_6months)

**Advice from the patient’s treating doctor that a reduction in visits was safe and that they were suitable for patient-led surveillance**

- “Due to the quantity of moles I have, I find regular personal checking and doctor checking helpful for my treatment, I am also willing to use the mole scope process to reduce doctor’s visits if I am deemed suitable.” (025_intervention_6months)
- “Only acceptable if specialist/doctor gave strong assurance that reduced visits were appropriate.” (085_intervention_6months)

**Why a reduction in routinely scheduled visits would not be acceptable.**

**Reassurance from being checked by physician with specialist expertise**

- “Specialist knowledge practised daily is expertise that should be lifesaving. In my opinion there is no substitute for this type of medical examination. Therefore, I would not reduce my medical check frequency.” (030_control_6months)
- “Whilst confident I could spot a change, I would still like reassurance of a specialist.” (088_intervention_baseline)
- “They are the experts in their fields, not me. I can see, what I think are changes to moles, but they know and when you have had Melanoma you want to be sure.” (026_control_baseline)
- “The expert eye of the specialist would be missed.” (043_control_6months)
- “I like the reassurance of regular specialist visits. I have so many moles it is hard to keep track with certainty. Do not visit GP for skin checks, only the specialist.” (088_intervention_6months)
- “I regard my annual check-up as a 'safety net' of sorts and get some assurance from having a specialist observe my skin for any changes/concerns.” (096_intervention_baseline)
- “Having had two melanomas, I feel comfort in having my skin routinely checked by a specialist. I am comfortable checking my own skin and am happy to raise concerns if needed, but I would want to continue with my routine scheduled visits.” (062_intervention_baseline)

**Perceived very high risk of a subsequent melanoma **

- “No circumstances would be acceptable to me, I have a very huge number of dysplastic nevi and had 2 melanomas removed and an area of moles pre melanoma removed. Due to the large number, I feel regular scheduled visits must continue.” (003_intervention_6months)
- “I’m a high-risk melanoma patient after having 4 melanomas removed. I will always see my specialist 1 to 2 times per year for the rest of my life.” (081_control_6months)
- “One needs regular scheduled visits to keep on top of health issues, especially as one ages.” (049_control_6months)
- “Because of family history I would not feel confident having myself or other family members check my skin - especially in areas that I can't see on my body.” (031_intervention_6months)
- “I adopt a ‘belts and braces’ approach. Any missed chance could be fatal for me.” (030_control_baseline)

**Low confidence in their ability to undertake thorough skin self-examination and detect a concerning lesion**

- “The anxiety of not having regular appointments in case I have missed something in my checks.” (045_intervention_6months)
- “Prefer regular check-ups with my melanoma specialist as I’m not confident enough with my own recognition.” (022_intervention_6months)
- “Not confident enough at present that I can recognise a melanoma.” (008_control_6months)
- “I would like to be active in the process and take responsibility, but I don't feel comfortable - I am an anxious person and I feel inadequately prepared to monitor it solely on my own.” (015_control_baseline)
- “I would worry I would miss something in my checks and then the longer between doctor visits the more advanced the melanoma becomes.” (045_intervention_baseline)

**Need for minimum annual clinic review**

- “I only see the Melanoma clinic 1 per year now so I would not like to go any less than this. Once per year is not onerous.” (014_control_6months)
- “I need the reassurance that I get from a yearly visit with the specialist” (063_control_6months)
- “I see a specialist now every 12 months (last 4 years every 6 months). Every 12 months is not an issue, and it gives me the confidence that I am being examined by a professional.” (004_control_baseline)
- “It's always nice to know that an expert will look at your skin at least once per year, even if you yourself are highly conscientious in checking your own skin.” (093_control_6months)
- “I am on yearly checks now, reducing this may make me worry more that something may be missed.” (071_intervention_baseline)

### Circumstances in Which a Reduction in Routinely Scheduled Visits Would Be Acceptable

Trial participants reported prerequisites for a reduction in routinely scheduled visits to be acceptable. These included sufficient time had passed without any subsequent new melanomas, if patients could access expert advice from their specialist via teledermatology and in-person checks could be arranged quickly, if the patient had confidence that patient-led surveillance using digital technology was effective for them personally (answers from control patients were hypothetical in nature, but intervention patients reported that after using the intervention, they were more confident that digital technology tools could help them identify a melanoma early), and if they were reassured that a reduction in routinely scheduled visits was safe and suitable for them.

### Why a Reduction in Routinely Scheduled Visits Would Not Be Acceptable

Other trial participants provided explanations for why a reduction in routinely scheduled visits would not be acceptable to them. These included patients who perceived or felt that they were at very high risk of developing a subsequent melanoma; patients who valued the expertise of their specialist and felt reassurance from being checked by a physician with specialist expertise, even if they were confident in SSE; and patients with low confidence in their ability to undertake thorough SSE and detect a concerning lesion. Some patients also said that they were attending annually scheduled reviews and that a further reduction would not be acceptable; others who were attending more frequently said that a partial reduction to annual visits may be acceptable but not any less frequent.

### Why Reduction in Routinely Scheduled Primary Care Visits May Have Been More Acceptable Than a Reduction in Scheduled Specialist Visits

Some participants expressed an explicit preference for being checked by their specialist skin doctor (dermatologists and primary care physicians with training in skin cancer) over their local primary care physician, explaining that they had trust in the expertise of their specialist. Other patients explained that only their specialist, not their local primary care physician, played a significant role in their melanoma care (Textbox 4).

**Why reduction in routinely scheduled primary care visits may have been more acceptable than a reduction in scheduled specialist visits (note, general practitioner [GP] in Australia is equivalent to primary care physician in North America).**

**GP not involved in patient’s melanoma care**

- “I only go to GP for suture removal. I do monitor my skin and if an issue comes up, I contact the melanoma clinic.” (061_control_6_months)
- “With my family history I have annual check. But if anything worries me I photo and email or book a short notice check. My GP says I’m in the best hands and doesn’t get too involved.” (064_control_6_months)
- “My GP is happy to leave these inspections to my skin Dr.” (008_control_baseline)
- “I never consult my GP for skin concerns.” (063_control_baseline)
- “I only see my GP when necessary and this isn't connected with my skin examinations.” (002_intervention_6_months)
- “Do not visit GP for skin checks, only the specialist.” (088_intervention_6_months)
- “I have routine specialist check annually and feel this should continue. Following Molescope photo submission I have been advised to get a mole checked urgently - GP did not want to touch it...” (013_intervention_6_months)

**Trust in skin specialist over GP**

- “GPs are good however I think you need to have found a GP that you are confident in and have a good relationship with that you can leave the office with either a plan or similar level of confidence that you leave a Dermatologist office with.” (072_control_6_months)
- “...don't have confidence in my GP (they just send me to Melanoma specialist) or in my own detection skills.” (066_control_baseline)

## Discussion

In this pilot trial of 100 patients randomized to patient-led surveillance or usual care, we found no difference between randomized groups in the acceptability of a reduction in routinely scheduled visits. At 6 months after randomization, 34% reported that a reduction would be at least slightly acceptable, 32% reported that a reduction would not be at all acceptable, and 34% did not respond to the question. Among those identifying that a reduction in routinely scheduled visits could be acceptable, a number of prerequisites were identified: sufficient time without a new primary melanoma or recurrence; an unscheduled appointment could be made at short notice if the patient noticed something concerning; their melanoma doctor advised that reducing visit frequency was suitable for them; and they had confidence that an alternative method of surveillance, such as patient-led surveillance, was a safe and effective alternative to usual care.

Our findings agree with previous reports that while some patients may be willing to reduce the frequency of routinely scheduled visits if this is recommended by their clinician [1], many patients, and especially high-risk patients, may be reluctant to do so [9]. The frequency of routinely scheduled clinic visits varies across settings and clinicians and is often influenced by local clinical culture [10,11]. The “less is more” approach [12] aims to deimplement or deadopt inappropriate health care, including that which is untested [13-15]. This may be difficult when the potentially inappropriate care is the usual and expected care and if the new intervention requires learning complex, technical skills that differ significantly to those of the existing health care [13]. Other barriers to deimplementation include patient fear and anxiety, and overestimation of the effectiveness of usual health care by both patients and clinicians [16]. Patients at risk of a new primary or recurrent melanoma may experience reassurance from routine visits, even if it may be otherwise unnecessary. Less contact with their specialist skin doctor may also potentially weaken their patient-doctor relationship [13].

For some patients, taking on more responsibility for surveillance may be empowering and lead to improved clinical and psychological outcomes, especially if they also have an effective skin check partner to support them [17]. Others may not want to take on this responsibility and prefer to continue with their usual routinely scheduled clinic visits [1,9].

Study limitations include missing data (34% [34/100] did not complete the 6-month questionnaire) and unknown generalizability of the trial population. Patients are more likely to be receptive to decreases in visit frequency if there is clear evidence that alternative models of surveillance are safe and effective and that these alternatives do not mean a reduction in care but rather higher value care [10]. A larger MEL-SELF RCT that is currently underway will generate further evidence on the acceptability of a reduction in routinely scheduled visits when undertaking patient-led surveillance [18].

## Appendix

Multimedia Appendix 1 Melanoma Self Surveillance (MEL-SELF) pilot randomized clinical trial protocol.

Multimedia Appendix 2 Survey questions relating to hypothetical reduction in routinely scheduled clinic visits.

Multimedia Appendix 3 Flow of participants: Melanoma Self Surveillance (MEL-SELF) pilot randomized clinical trial.

Multimedia Appendix 4 Exploratory subgroup analysis by American Joint Committee on Cancer substage.

Multimedia Appendix 5 CONSORT-EHEALTH (V 1.6.1) - Submission_Publication Form.

## Abbreviations

AJCC: American Joint Committee on Cancer substage
CONSORT-EHEALTH: Consolidated Standards of Reporting Trials of Electronic and Mobile Health Applications and Online Telehealth
MEL-SELF: Melanoma Self Surveillance
RCT: randomized clinical trial
REDCap: Research Electronic Data Capture
SSE: skin self-examination
