# Supplementary material for: Acceptability of a Hypothetical Reduction in Routinely Scheduled Clinic Visits Among Patients With History of a Localized Melanoma (MEL-SELF): Pilot Randomized Clinical Trial
Source: JMIR Dermatol. 2023 Jun 26;6:e45865. doi: 10.2196/45865 (PMC10335154; doi:10.2196/45865)
Supplement: Multimedia Appendix 1 [file derma_v6i1e45865_app1.pdf]

02.17 MEL-SELF

A pilot randomised controlled trial of patient-led surveillance compared to clinician-led surveillance in people treated for localised melanoma

Registration number: ACTRN12616001716459
Version 6.0 dated 22 May 2020

Principal Investigator: Associate Professor Katy Bell
Address and contact details: Sydney School of Public Health | Sydney Medical School
Room 131 Edward Ford Building A27
Mobile: +61 415764321
Email: katy.bell@sydney.edu.au

Signature: ________________ Date: 22 May 2020

For information related to the Trial Management Committee membership please refer to the Operations Manual. This is an independent investigator initiated co-operative group trial.

Administration

Sponsor:	The University of Sydney
Collaborative/Coordinating Group:	Melanoma and Skin Cancer Trials<br>MASC Trials<br>The Poche Centre<br>40 Rocklands Road<br>North Sydney NSW 2060 Australia<br>Telephone: +61 2 9911 7294<br>Fax: +61 2 9954 9435<br>Email: anzmtg0217@melanoma.org.au

Document Version History

Version	Version date	Main Changes
2.0	30/08/2017	Initial protocol
3.0	23/04/2018	- ANZMTG added as lead collaborative group/co-ordinating centre<br>- ASICA smartphone App replaced with web-based version of the ASICA skin checker App<br>- Duration of treatment/study updated<br>- Schedule of assessments: Time and Events table added<br>- SMS text added as a patient reminder option
4.0	24/01/2019	- Australia and New Zealand Melanoma Trials Group (ANZMTG) is now known as Melanoma and Skin Cancer Trials Ltd. (MASC Trials). MASC Trials is now the lead collaborative group and co-ordinating centre<br>- Recruitment process updated for in clinic recruitment and not a bulk mail out<br>- Baseline questionnaires will be a part of the study invitation package<br>- Teledermatologist case review recommendations have been updated
5.0	3/12/2019	- Confidentiality section: Data is stored at University of Sydney
6.0	22/05/2020	- 8.6.2 End of Study updated from 6 months to 12 months

1. Contents

1. Contents ... 4
2. Abbreviations: ... 6
3. Study synopsis ... 7
4. Project Team Roles and Responsibilities ... 8
5. Introduction and rationale ... 8
   5.1. Background ... 8
   5.2. Justification/ Significance ... 9
   5.3. Lay summary ... 9
6. Study design ... 9
   6.1. Research question(s)/ aim(s): ... 9
   6.2. Study design ... 10
7. Study population ... 10
   7.1. Inclusion Criteria ... 10
   7.2. Exclusion Criteria ... 10
8. Study procedures ... 11
   8.1. Enrolment procedure ... 11
   8.2. Stratification and Randomisation ... 11
   8.3. Interventions/ Groups ... 11
   8.4. Blinding ... 12
   8.5. Duration of treatment/ Study ... 12
   8.6. Assessment schedule and follow up ... 12
   8.6.1. Follow-up questionnaire (T1): 6 months ... 13
   8.6.2. End of study. ... 13
   8.6.3. Withdrawal and Lost to Follow up ... 13
9. Data Collection ... 13
10. Intervention ... 14
   10.1. Intervention and comparison arms ... 14
   10.2. Intervention/ treatment modification ... 15
   10.3. Distribution, packaging and labelling ... 15
   10.4. Accountability for intervention ... 15
11. Safety Reporting ... 15
   11.1. Adverse Events ... 15
   11.2. Serious Adverse Events ... 15
12. Outcomes/ Measures ... 16
   12.1. Primary outcome ... 16
   12.2. Secondary outcomes ... 16
   12.3. Potential confounders and biases ... 17
13. Statistical methods ... 17
   13.1. Sample size and justification ... 17
   13.2. Feasibility ... 17
   13.3. Early stopping ... 18
   13.4. Analyses plan ... 18
      13.4.1. Statistical analysis for primary endpoint ... 18
      13.4.2. Statistical analysis for secondary endpoints ... 18

13.5. Secondary analyses 18
13.6. Missing data 19
13.7. Deviations from original statistical plan 19
14. Responsibilities of the Principal Investigator at each site 20
15. Reporting of Results 20
16. Quality Assurance 21
16.1. Data Management and Source Data Verification 21
16.2. Protocol Amendments 21
16.3. On Site Monitoring 21
16.4. Site Audits 21
16.5. Confidentiality 21
17. References 21
18. Appendices 22

2. Abbreviations:

AE	Adverse event
AJCC	American Joint Committee of Cancer
ANZMTG	Australia and New Zealand Melanoma Trials Group
App	Smartphone Application
ASICA	Achieving Self-Directed Integrated Cancer Aftercare
CI	Co-investigators
CR	Complete response
CRF	Case report form
CTCAE	Common terminology criteria for adverse events
DASS-21	Depression Anxiety and Stress Scales
DSMC	Data Safety Monitoring Committee
FCR	Fear of Cancer Recurrence
FCRI	Fear of Cancer Recurrence inventory
FDA	Food & Drug Administration
GCP	Good Clinical Practice
GP	General Practitioner
HREC	Human research ethics committees
ICF	Informed consent form
ICH GCP	International Conference on Harmonisation of Good Clinical Practice
ID	Identification
IELab	Australian Industrial Ecology Virtual Laboratory
Kg	Kilogram
M	Million
MASC Trials	Melanoma and Skin Cancer Trials Ltd.
MM	Millimetre
MIA	Melanoma Institute Australia
NMSC	Non-Melanoma Skin Cancers
RCT	Randomised Controlled Trial
SEIFA	Socio-Economic Indexes for Areas
SSE	Skin Self-examination
TMC	Trial Management Centre
USYD	University of Sydney

3. Study synopsis

Title:	A pilot randomised controlled trial of patient-led surveillance compared to clinician-led surveillance in people treated for localised melanoma
Study Number:	02.17 MEL-SELF
Primary Objective:	1. To assess the feasibility of patient-led surveillance.
Rationale:	Most new (and recurrent melanomas) are detected by patients or family members in between scheduled visits, and this might be increased with additional training and support for patient skin self-examination and with Teledermatology assessment of patient-detected abnormalities. This may enable less frequent scheduled follow-up with melanoma clinicians. Fewer scheduled visits may be just as safe, cause less anxiety and consume fewer resources.
Study Design:	Randomised Controlled Trial (RCT)
Sample Size (by treatment group):	50 participants allocated to the intervention group and 50 participants allocated to the usual care group
Study Arms (Intervention and Control):	Intervention group:<br>(i) Patient-led surveillance: web-based questionnaire based on the ASICA skin checker App (with instructional videos and electronic reporting of lesions discovered on skin self-examination), Smartphone App for participants to perform teledermoscopy with feedback to participants of Dermatologist report, email/SMS reminders to perform total skin self-examination every 2 months;<br>(ii) An educational booklet.<br>(iii) Usual scheduled follow-up;<br>(iv) Unscheduled visits as needed<br><br>Control group:<br>(i) an educational booklet<br>(ii) Usual scheduled follow-up ;<br>(iii) Unscheduled visits as needed
Study Population	Adult patients who are undergoing routine melanoma skin cancer surveillance.
Study Endpoints (Primary and Secondary):	1. Primary outcome: Proportion of patients who are contacted and invited to participate, who are finally randomised in the trial.<br><br>2. Secondary outcomes: Adherence with recommended skin self-examination practice (total body self-examination conducted two- monthly), thoroughness, confidence, knowledge attitudes and beliefs about skin self-examination, fear of new or recurrent melanoma, general anxiety, stress and depression, number of lesions surgically excised, number of follow up visits attended (both scheduled and unscheduled), inter-dermatologist reliability of teledermoscopy, resource use and costs (both financial and carbon emission costs), and results from the qualitative sub-study.

4. Project Team Roles and Responsibilities

Study Chair:
Dr Katy Bell, Senior Lecturer in Clinical Epidemiology, Public Health, School of Public Health, The University of Sydney.

Protocol Development Group:
Professor Monica Janda (academic health psychologist)
Associate Professor Anne Cust (cancer epidemiologist)
Associate Professor Robin Turner (statistician)
Professor Les Irwig (clinical epidemiologist)
Associate Professor Pascale Guitera (academic dermatologist)
Associate Professor Robyn Saw (melanoma surgeon)
Associate Professor Victoria Mar (academic dermatologist)
Professor Peter Soyer (academic dermatologist)
Dr Mbathio Dieng (epidemiologist & health economist)
Dr Jolyn Hersch (academic health psychologist)
Associate Professor Rachael Morton (melanoma health economist)
Mr Donald Low (consumer investigator)
Ms Cynthia Low (consumer investigator)
Ms Amelia Smit (public health researcher)
Ms Mai Nguyen (health economist)

5. Introduction and rationale

5.1. Background

The increasing incidence of melanoma in Australia (~500% increase from 3,526 new melanomas in 1982 to 17,570 by 2020)[1] is largely driven by increased early detection of localised melanoma before it has spread from the skin[2]. After treatment, these patients are at risk of developing a recurrent or new primary melanoma and/or NMSCs and are recommended to undergo lifelong follow-up at intervals based on stage that range between 3 to 12 months[3]. However, they have a very good prognosis in terms of life expectancy: the same as the general population for melanoma in situ (stage 0), and 96% 20 year survival for thin melanomas (<1 mm) (together these are >80% of all localised melanomas[4]). The potential benefits of clinician surveillance for recurrent or new primary melanoma and NMSC in ensuring timely treatment, needs to be balanced against costs and possible physical and psycho-social harms of frequent scheduled follow-up clinic visits and investigations [5,6]. The costs of follow-up are substantial and have been estimated at AU$44M over 5 years for American Joint Committee of Cancer (AJCC) stage I/II[7]. There are also opportunity costs in terms of clinician time, with waiting lists for new patients in public dermatology clinics potentially getting longer. Fewer scheduled follow-up visits may have little impact on the detection of recurrence and new primaries[8] and can result in substantial cost savings[9]. Clinicians are more likely to choose this option if they are confident in the patient's ability to do their own surveillance through skin self-examination (SSE) [10].
Currently there is no evidence that frequent scheduled follow-up has any effect on patient survival[11]. In contrast, there is evidence that self-detection of recurrence is associated with higher survival compared to detection by a clinician, highlighting the importance of patient vigilance. Yet despite SSE being universally recommended by clinical guidelines, education and practice remains suboptimal, with less than half of respondents in a study of melanoma patients in NSW [paper currently under review], and only 14% in an American study[12], indicating that they examined all parts of the body. In fact current evidence suggests that few people carry out SSE thoroughly, with many failing to view hard-to-see areas, seeking assistance by a partner, or documenting lesions that they need to check again to notice changes[13].

5.2. Justification/ Significance

This pilot study is the first to evaluate the feasibility of patient-led surveillance through: Web-based version of the 'ASICA skin checker' App (instructional videos, guided self-examination and electronic reporting), Smartphone App using teledermatoscopy of patient detected lesions (detailed images transmitted electronically for dermatology review), email + SMS reminders + educational booklet + scheduled and unscheduled clinic visits. It will support planning for a large trial to compare the effects of patient-led surveillance and clinician-led surveillance on health outcomes, psychological outcomes and resource use. The larger study will aim to demonstrate that patient-led surveillance results in health outcomes that are at least as good as clinician-led surveillance, with better psychological outcomes and less health resource use. Our work will allow for evidence-based follow-up after treatment of localised melanoma, to maximise patient wellbeing and the early detection of new or recurrent melanoma, while minimising costs to the health system.

5.3. Lay summary

Background and Aim: Most melanomas are detected by patients or family members between scheduled visits; Even more might be detected if patients are trained in how to self-examine their skin and have access to timely dermatology review. Frequent follow-up of localised melanoma is time and resource intensive and has not shown improved outcomes. This pilot study, and the larger one that will follow, aim to provide evidence on the best model of follow-up care after treatment for localised melanoma.

Research Design: This proposal is for a pilot randomized controlled trial among patients who have had surgery for localised melanoma and are followed up at recruiting skin cancer specialist clinics or general practice clinics. We will compare the effects of offering patient-led surveillance (Web-based version of the ASCIA skin checker + dermatoscope + Teledermatology + email and text reminders to support self-examination + an educational booklet + scheduled and unscheduled clinic visits) to clinician-led surveillance (an educational booklet + scheduled and unscheduled clinic visits). We will document how well participants are able to perform self-examination, their levels of melanoma-related anxiety, the number of skin lesions biopsied or removed, and the costs of follow-up to the participant, to the healthcare system, and to the planet (in the form of carbon emission costs). We will recruit 100 people who have been treated for a first primary localised melanoma, own a smartphone, and are being followed up at a recruiting skin cancer specialist clinic or general practice clinic. Potential participants will be screened in clinic by the treating clinician. Potential participants will be given the study invitation package either in clinic or by mail out. Patients who are found to be eligible and who consent to participate will be randomised to patient-led surveillance or clinician-led surveillance. Participants will be sent questionnaires at six months to measure outcomes using paper or online surveys. We will also collect data through the web-based version of the ASICA skin checker (in the intervention group), patient diaries (to measure out of pocket costs), and from the clinic databases (including follow-up visits, surgical procedures and pathology reports of any skin lesions removed).

6. Study design

6.1. Research question(s)/ aim(s):

The **primary aim** of the study is to investigate whether patient-led surveillance (Web-based version of the ASICA skin checker + smartphone App and dermatoscope attachment + Teledermatology + email/SMS reminders) is feasible.

The **secondary aims** are to investigate whether patient-led surveillance:

1. increases patients' adherence with recommended SSE practice (total body self-examination conducted two- monthly)
2. improves confidence in, knowledge of, attitudes and beliefs about, skin self-examination,
3. decreases level of fear of new or recurrent melanoma, general anxiety, stress and depression,

4. decreases the number of lesions surgically excised, the number of follow up visits attended (both scheduled and unscheduled), resource use and costs and carbon emissions (costing study)
5. Increases acceptability of reducing scheduled clinic visit frequency
6. has acceptable inter-rater reliability for the Teledermatology assessment (reliability sub-study)
7. is accepted by clinicians and patients views (qualitative sub-study)

6.2. Study design

This study is a 2-armed RCT with 1:1 allocation to intervention vs the control group, with a Teledermatology repeatability sub-study nested within the intervention arm to estimate the inter-rater reliability of Teledermatology.

In addition, we will conduct a costing study to estimate the total costs to the health system, to patients, and to the planet (carbon emissions) for the intervention and control groups. All costs associated with SSE, skin surveillance and management of newly identified lesions, out of-pocket costs and opportunity costs will be included. We will also measure and value all resources used (micro-costing approach) to estimate the carbon emissions associated with melanoma surveillance for the intervention and control groups.
We will conduct a qualitative sub-study to understand patients' and clinicians' acceptability and satisfaction with the intervention and explore components which may need to be changed. The qualitative evaluation will be in the form of phone or face to face interviews at the end of the 6 month follow-up.

7. Study population

We will recruit adult participants who have been treated for a first primary melanoma, stage 0/I/II, and who are undergoing regular melanoma follow-up at recruiting melanoma and skin cancer clinics in NSW.

This pilot study aims to recruit 100 patients from the Melanoma Institute Australia (MIA) and Royal Prince Alfred Hospital (RPAH) (specialist treatment centres) and the Newcastle Skin Check clinic (GP run clinic). Recruitment will be done over six months.

7.1. Inclusion Criteria

- Patients treated for stage 0/I/II melanoma who are attending regular melanoma follow-up as indicated by scheduled or recommended visit within next 12 months.
- Are able to self-examine;
- Have a suitable study partner (spouse, partner, family member, friend) to help with self-examination
- Own a compatible Smartphone (and have access to Wifi / email / SMS text messaging);
- Are able to give informed consent;
- Have sufficient English language skills to read the materials and complete the questionnaires;
- Aged 18+ years old.

7.2. Exclusion Criteria

- unable to perform self-examination
- Patient who currently or previously have had stage III/IV melanoma
- Patients with a known past or current diagnosis of cognitive impairment

8. Study procedures

8.1. Enrolment procedure

All patients treated for localised melanoma who are attending regular follow-up, will be identified in clinic by the clinician. The potential participant will be contacted by a research coordinator who will explain the study and give the patient an invitation package either in person or by mail. The initial study invitation package includes: Study Invitation Letter, Participant Information Sheet, Consent Form, Participation Card, Baseline Questionnaire (to measure demographics, knowledge, attitudes and confidence on self-examination and smartphone Apps, and their baseline level of fear of new or recurrent melanoma (using a melanoma specific version of the Fear of Cancer Recurrence Inventory Severity subscale)[14] and reply paid envelope. The letter of invitation will be printed on the clinic letterhead and will be signed by patients' treating clinician, preferably as original signatures but alternatively using electronic signatures with their permission. Participants will be able to opt into the study by returning the signed consent form and participation card to the researchers by reply paid mail or to decline study participation by returning the participation card, or by phone or email. Potential participants will be asked to indicate whether they meet each of the inclusion criteria (e.g. own a compatible Smartphone etc.) and to specify on their participation card how they would like to receive the study dermatoscope and questionnaires reminders (on Text SMS or email). Participants who do not return their consent form or participation card within two weeks will be contacted via telephone by the research staff. If the participant does not return their consent form after this telephone call, no further contact will be made. Participants that do not respond will be recorded as screen fails. The clinic staff will be informed which patients have agreed to participate in the study.

Consenting participants will then be sent a baseline package in the mail.

8.2. Stratification and Randomisation

Patients will be stratified by the following factors:

- AJCC Melanoma Stage (0, IA, IB, IIA, IIB/IIC)
- Sex/gender (male/female)
- Age group (18–40, 40–60, 60+)
- Clinic type (GP clinic/specialist clinic)

Patients will be randomised 1:1 taking into account the above listed stratification factors using a randomisation to one of two study arms:
Randomisation will be performed using the offsite randomisation service provided by the NHMRC Clinical Trials Centre, The University of Sydney, thus ensuring allocation concealment. The research coordinator will call the NHMRC Clinical Trials Centre (using their telephone randomisation service) to randomise the patient once a consent form and the baseline questionnaire has been received. Consenting patients who meet all inclusion/exclusion criteria will be randomised in a ratio of 1:1 to the intervention or usual care.

Full instructions on how to randomise patients are described in the Operations Manual.

8.3. Interventions/ Groups

<u>Participants allocated to the intervention will receive</u>:

- An educational booklet 'Your guide to early melanoma',
- Web-based ASICA skin checker log-in. The web-based ASICA skin checker combines instructional videos, guided total skin self-examination and electronic reporting,[15]
- A dermatoscope to attach to their phone similar to that reported in *Manahan et al.*[16]*.*
- Detailed written instructions on how to use the smartphone App and dermatoscope.

- Video outlining how to use the dermatoscope and associated App/online submission platform.
- Email or SMS text reminders every 2 months to perform SSE.
- Teledermoscopy: participants are required to take dermoscopic images of at least 3 lesions and up to 9 lesions on each SSE, including all lesions that concern them, and submit these through the smartphone App. They are also required to enter a text description of the results of SSE for each part of the body and submit this through the web-based ASICA skin checker. Both dermoscopic images and text reports will be sent securely to one of the study dermatologists, who will undertake teledermatology review within 3 days and feedback the results to the participant.
- Unscheduled visits as needed in addition to those prompted through Teledermatology.
- Scheduled visits as per treating clinician.

<u>Those allocated to the control arm will receive:</u>

- An educational booklet 'Your guide to early melanoma';
- Unscheduled visits as needed
- Scheduled visits as per treating clinician

All participants will receive two study questionnaires; one at baseline and one at 6 months.

8.4. Blinding

Blinding of participants, site staff and treating clinicians will not be possible in this trial. Where possible, outcome assessment will be done masked to study group allocation.

8.5. Duration of treatment/ Study

Unless the study is closed prematurely, it will proceed to the pre-defined accrual target. Following announcement of closure to recruitment by the research team, no further enrolment will be possible. It is anticipated, based on estimates from individual sites that the accrual target will be reached over a period of 6 months. Each participant will be followed up for 6 months. The timeline below provides an outline of anticipated study milestones:

**Timeline**	**2017**				**2018**				**2019**				**2020**			
Apply for ethics approval and preparation of study materials																
Recruitment & random allocation																
6 month follow-up data collection and entry																
Analysis of 6 months follow up data finalised and paper drafted																
Qualitative phone interview study																
Costing study																
Data cleaning, statistical and cost analyses																
Paper & Dissemination																

8.6. Asses

sment schedule and follow up

Follow-up: All participants will be complete a questionnaire at baseline (T0: prior to randomisation) and 6 months (T1) follow-up which they can complete in hard copy or online. Data will be extracted from clinic databases for 6 months from randomisation, including surgical procedures and histopathology.

8.6.1. Follow-up questionnaire (T1): 6 months

Participants will be mailed the first follow-up questionnaire (or provided with a link to the online survey via email) 6 months after randomisation. If participants do not return their completed questionnaire within 1 week of postage or email, the researchers will contact the participant by SMS text and/or email (according to the participant's preferred method of contact) as a reminder about the study. The researchers will remind the participant 5 times. If there are delays with the return of the questionnaire, and the participant appears to have problems completing the questionnaire on paper or online, the research coordinator will offer to administer the questionnaire over the telephone.

8.6.2. End of study.

Measurement of outcomes will be at 6 months (T1questionnaire) for evaluation of intervention effects. If a patient has not completed the schedule of follow up visits, further follow up of outcomes will continue up to a maximum of 12 months. If a patient withdraws consent to participate, a Study Discontinuation Form will be completed (see below).

Similarly, if a patient dies, a Study Discontinuation Form will be completed and a copy of the death certificate or discharge summary provided. Reason for death, melanoma/skin cancer related or not, will be considered in the final analysis.

8.6.3. Withdrawal and Lost to Follow up

Participation in this study is voluntary; patients are able to withdraw at any time.

If a patient decides to stop their follow-up visits but is willing to keep in contact via telephone, their health status will be periodically ascertained by way of phone contact with their general practitioner or by direct phone contact with the patient.

For those patients who want to have no further contact, all correspondence regarding the trial will be discontinued. A Study Discontinuation Form is to be completed at the last study visit the patient attends and this is to be submitted to the Trial Coordinating Centre.

The National Death Index at the Australian Institute of Health and Welfare and other local databases, including clinic databases, may be used to collect outcome information on patients who have been lost to follow-up.

9. Data Collection

Trial data will be recorded in full on the CRFs (hardcopy to each site) or eCRFs (eDatabase).

The site is required to complete all appropriate data entry fields as specified on the forms. The investigator will be asked to confirm the accuracy of completed CRFs by signing forms as indicated. Source documents pertaining to the trial must be maintained by investigational sites. Source documents may include a subject's medical records, hospital charts, operation reports, the investigator's subject study files, as well as the results of diagnostic tests such as laboratory and pathology results.

The Trial Management Centre (TMC) may request copies of some source documents in support of the CRF as a quality assurance exercise. All study-related documentation is required to be maintained by the site for 15 years following completion of the study.

10. Intervention

10.1. Intervention and comparison arms

The intervention comprises:

- **The web-based ASICA (Achieving Self-directed Integrated Cancer Aftercare) Skin-Checker** is used to record regular Skin Self-Examination.[15] The patient will be prompted to perform a skin exam every two months. When the patient discovers a skin change or lesion, the patient will report the changes on the web-based ASICA survey. The skin report contains a description of the change or lesion and where it is located. Participants are required to provide text reports on each part of the body before they can proceed with submitting the report through the site (if there are no concerning changes/lesions then this is reported for each part of the body).
- **Dermatoscope and Teledermatology**: Smartphone App and dermatoscope that can be attached to a smartphone for patients to use. The device allows magnified images of skin lesions to be taken under polarized light for electronic transmission to a specialist. This store and forward process is called "mobile teledermatology". Three dermatologists will be rostered on to review the reports and images of patient detected lesions, and feedback recommendations to the patient as to the action that needs to be taken. They will report results within 3 working days of receiving the electronic text report and images from the patient. If the report is not reviewed in this timeframe the site study coordinator will notify the patient of a delay in reporting. They will provide one of the following recommendations:
    - Follow Up Photo - There is no immediate concern today, but this case should continue to be monitored. Please take another picture and resubmit for review.
    - Retake Photo - Unfortunately, the image quality is not sufficient for teledermatology review. Please retake the photo.
    - Clinical Exam - We recommend that you book an appointment with your doctor to examine this case. Please call your melanoma clinic to make an appointment at your earliest convenience.
    - No Concern Today - This case does not seem to be suspicious at this time. Self-monitor and review with your doctor at your next scheduled visit.
    - Biopsy/Excision - We recommend that you book an appointment with your doctor to examine this case as biopsy or excision may be needed. Please call your melanoma clinic to make an appointment at your earliest convenience.
    - Other
- Educational booklet 'Your guide to early melanoma': The freely available booklet developed by the Melanoma Institute Australia for patient support and comprises a treatment-management organiser and detailed information source in one booklet. Contents include: - Diagnosis summary - Melanoma glossary - Treatment plan - Pain diary - Medication chart - Body maps for self-examination - Frequently asked questions - Supportive services - Information on treatments and surgical procedures
- Written and video instructions on how to use the dermatoscope + Smartphone App
- Email and/or SMS text reminders every 2 months to perform self-examination
- Unscheduled visits as needed in addition to those prompted through Teledermatology
- Scheduled visits as needed
- Monthly diaries capturing clinic visits and travel costs

The control arm comprises

- An educational booklet 'Your guide to early melanoma';
- Unscheduled visits as needed

- Scheduled visits as needed
- Monthly diaries capturing clinic visits and travel costs

10.2. Intervention/ treatment modification

Participation in this study does not stop participants in either the intervention or control groups from seeking professional medical care. Access to formal health services will, however, be recorded as part of the study.

10.3. Distribution, packaging and labelling

The initial Invitation letter will be sent on clinician letterhead to the participant at the address on file at the clinic database. Following consent and receipt of the baseline study questionnaire, the study packages will be distributed by the research coordinator, according to their random intervention or control group allocation, to participants through the mail or during their routine clinic visit (as directed by participant preference).

10.4. Accountability for intervention

It is the responsibility of the research coordinator to make sure that all participants receive their packages according to their randomised group allocation.

11. Safety Reporting

11.1. Adverse Events

An Adverse Event (AE) can be any unfavourable and unintended sign, symptom, or disease temporally associated with the use of an intervention, whether or not related to the intervention.

In cases of surgical or diagnostic procedures, the condition/illness leading to such a procedure is considered as the AE rather than the procedure itself.

Medical device adverse event / incident:

- An event associated (caused or partially attributable) with the use (or misuse) of a medical device.
- An event that resulted in or could have resulted in (had effective intervention not taken place) serious injury, illness or death to patient, healthcare worker or other person.
- Faults that may affect the quality of device, timeliness of assessment and cost-effectiveness such as, problems with getting the device to operate, repeated repairs, device design and difficulty of use.

All AEs with the smartphone App and dermatoscope will be recorded and reviewed by the trial management committee.

11.2. Serious Adverse Events

SAE Definitions

A serious adverse event (SAE) is any untoward medical occurrence which:

- is fatal;
- is life-threatening;
- requires unanticipated in-patient hospitalisation or prolongation of hospitalisation;
- results in persistent or significant disability or incapacity;
- is a congenital anomaly/birth defect;
- is a grade 4 (NCI CTCAE v4.0) toxicity;

and

- could be related to the device and/or software

The term “life threatening” in the definition of SAE refers to an event in which the participant was at risk of death at the time of the event; it does not refer to an event which, hypothetically, might have caused death if it were more severe.

SAE Reporting

All relevant and subsequent information relating to each reported SAE must be submitted on the CRF, along with any available source documentation, to the Trial Coordinating Centre. This must be completed within 24 hours of the site becoming aware of the event and all SAE CRFs require the signature of the Principal Investigator.

SAE Reporting Timelines

**Type**	**Timeframe**
Initial SAE Report	The site must submit the original SAE Form to the Trial Coordinating Centre within 24 hours of becoming aware of a trial participant experiencing an SAE. Please provide copies of relevant source documents if available. Should the site have any questions please immediately contact the Trial Coordinating Centre for assistance.
Updated Report	The site should provide any updates to the Trial Coordinating Centre as soon as possible. Please use the original SAE Form, and use additional Forms if more space is required. Please provide copies of all relevant source documents if available.
Completed Report	Once the event has resolved or if the participant has died, using the original SAE Form(s), the site should provide the complete report to the Trial Coordinating Centre as soon as possible. This must include relevant source documents.

All SAEs will be reviewed by the Study Chair and also summarised for review by the Data Safety Management Committee.

SAEs will be reported as per the local Ethics Committee (EC)/Research Governance Officer (RGO), in accordance with international and local laws and regulations.

Should any trial site have any questions they will immediately contact the Trial Coordinating Centre for assistance.

12. Outcomes/ Measures

12.1. Primary outcome

(*M1)* The primary outcome is the **proportion of patients who are invited to participate who are finally randomised into the trial**.

12.2. Secondary outcomes

(M2) Skin –Self Examination (SSE) including:

M2.1. *Adherence with recommended SSE practice* (total body self-examination conducted two-monthly); Participants will be asked how often they perform a complete examination of their skin.

M2.2. *Thoroughness, confidence, beliefs, attitude, and knowledge of SSE* will be assessed by items adapted from Janda et al.

(M3) Level of fear of new or recurrent melanoma (FCR) severity*:* FCR will be assessed using a modified (i.e. melanoma-specific) version of the 9 item Fear of Cancer Recurrence Inventory (FCRI) severity subscale, the most comprehensive multi-dimensional scale of FCR available.[7] A higher score is indicative of greater FCR.

(M4) General anxiety, stress and depression: Anxiety, stress and depression will be measured using the short version of the Depression Anxiety and Stress Scales (DASS-21).[17] The DASS-21 is a set of three 7-item self-report scales designed to measure the emotional states of depression, anxiety and stress. The depression scale measures dysphoria, hopelessness, devaluation of life, self-deprecation, lack of interest/involvement, anhedonia and inertia. The anxiety scale measures autonomic arousal, skeletal muscle effects, situational anxiety, and subjective experience of anxious affect. The stress scale assesses difficulty relaxing, nervous arousal, and feeling irritable and impatient.
***(M5)* Acceptability of reducing scheduled clinic visit frequency:** will be measured through item(s) designed specifically for this questionnaire.
***(M5)* Number of lesions surgically evaluated;**
***(M6)* Number of clinic visits attended** (both scheduled and unscheduled)
(M7) Inter-rater reliability for the Teledermatology assessment: We will carry out a repeatability study after trial close-out where the three study dermatologists independently report on cases they did not provide teledermatology assessment for during the trial. We will compare results across the three dermatologists to assess agreement.
(M8) Resource use*:* Patient out of pocket and health system costs associated with each arm of the trial will be estimated using a resource use diary. The diary will be used to document and measure health behaviours and service use - such as hospitalisation, other allied health consultations, use of alternative therapies and self-monitoring behaviours. The diary will also document days out-of-role (including paid and unpaid work), travel costs and carer costs. The diary will be based on existing resource use questionnaires resource use recorded in databases.
(M9) Qualitative results: We will carry out face-to-face or telephone interviews with a sub-set of intervention group participants at 6 months after randomisation to explore in depth patients' experiences with digitally supported self-examination. We will also interview clinicians to explore their experience of the benefits and limitations of the intervention.

12.3. Potential confounders and biases

(M10) Demographic and other risk factors including:
Age, sex, ethnicity, Indigenous status, language spoken, marital status, children, occupation, income, highest education level, postcode/SEIFA, brand of smartphone owned (if applicable), baseline use of digital technology/ internet, and personal history of depression or anxiety. We will collect these data using standardised items from the Australian Census questionnaire and other instruments, where appropriate.
Clinical characteristics of the index melanoma including AJCC sub-stage and site, retrieved from administrative datasets at the clinics.

13. Statistical methods

13.1. Sample size and justification

A sample size of 100 participants, with 1:1 allocation to intervention vs control groups, will ensure that the 95% confidence interval for the proportion of potential participants who are recruited has a margin of error of <10% (assuming that 30% of contacted, eligible participants are recruited).

A sample size of 30 participants in the qualitative sub-study is a well-accepted sample size in qualitative studies using in-depth interviews.[18]

13.2. Feasibility

Administrative data from our main clinic (MIA) indicates that approximately 800-900 patients are treated for localised melanoma at MIA each year and approximately 50% then attend regular follow-up at MIA.[19] Recruiting 100 patients over 6 months from all patients treated for localised melanoma who are attending follow-up at MIA and the clinic in Newcastle, is highly feasible.

13.3. Early stopping

There are no safety endpoints or criteria for early study termination.

13.4. Analyses plan

13.4.1. Statistical analysis for primary endpoint

The proportion of patients who are randomised into the trial will be estimated using the number of contacted patients as the denominator. The Wilson (score) method will be used to estimate the confidence interval for this proportion to assess the best and worst case scenarios supported by the pilot study data. We will investigate and describe any groups that had low participation rates in our pilot study.

13.4.2. Statistical analysis for secondary endpoints

We will use a chi-squared test to investigate the difference in proportion undertaking regular skin self-examination between the intervention arm and the usual care arm. A t-test will be used to compare fear of new or recurrent melanoma severity at 6 months between the intervention and usual care group. We will report any estimated differences (either proportions or means) with appropriate confidence intervals as the power for these comparisons will be limited.

13.5. Secondary analyses

Teledermatology Repeatability Study

Sub-study to assess Dermatologist diagnostic agreement of electronic text reports and images collected during the trial.

We will assess inter-rater agreement across three dermatologists, reporting on prospectively collected Teledermatology materials. After close-out of the trial, three dermatologists (CIs Guitera, Soyer and Mar) will independently review all electronic reports and images of patient detected lesions that they did not report on during the trial. All three dermatologists will report their results blinded to those of the other two dermatologists and to any other clinical information apart from that received during the Teledermatology (age and sex of patient and the text report describing the lesion where this was provided).

Agreement across the following broad clinical diagnostic categories will be calculated:

- No concern today [i.e. benign]
- Follow up photo [i.e. low probability of melanoma/NMSC]
- Clinical Exam [i.e. intermediate probability of melanoma/NMSC]
- Biopsy/excision [i.e. high probability of melanoma/NMSC]
- Only one category per patient episode allowed, where teledermatologist makes more than one diagnosis, most severe diagnostic category used.

We will construct cross-classification tables to separately compare inter-rater reliability for text report + macroscopic image and for text report + dermoscopic image. We will calculate percentage agreement and kappa statistics across diagnostic categories and 95% confidence intervals for these.

Costing study

We will identify, measure and value all resources used (micro-costing approach) to estimate the total costs to patients, to the health system, and to the planet (carbon emissions) for the intervention and control groups. All costs associated with SSE, skin surveillance and management

of newly identified lesions, out of-pocket costs, opportunity costs and carbon costs will be included.[20]

Patient diaries

Patient out-of-pocket and health system costs associated with each arm of the trial will be estimated using a monthly resource use diary.[21,22] The diary will be used to document and measure health behaviours and service use - such as hospitalisation, other allied health consultations, use of alternative therapies and self-monitoring behaviours. The diary will also record any melanoma follow up consultations and procedures conducted by other providers (e.g. GPs). We will ask patients to report the number of trips made to their melanoma follow-up clinic and the mode of transport used. We will also ask patients to report any visits to other physicians in the community for a skin lesion excision or other skin cancer related procedures. The time each patient spent to perform digital SSE and to attend follow-up visits will be recorded. The diary will also document carers' days out-of-role (including paid and unpaid work), travel costs and direct carer costs.

Review of medical records

All recorded melanoma follow up consultations and procedures will be extracted from the patients' medical records in the clinic databases. We will also extract all diagnostic tests during the trial period.

The mean (and standard deviation) volume of resource use by category and mean cost per participant will be tabulated. The difference in resource use and costs between the intervention and control groups will be presented with 95%CIs, and P- values for differences will be calculated by two sample t-tests.

Carbon Costs

We will then apply a carbon value to each of the types of resource use identified above. This will include direct estimation (e.g. travel) and indirect (e.g. life cycle costs of a tablet/smartphone). We will do this using data from the Australian Industrial Ecology Virtual Laboratory (IELab), which offers a unique platform for the compilation of customized economic input-output tables for the Australian economy. As a first step, we will couple these economic tables with data on carbon emissions for every economic sector. Next, we'll use these integrated tables to undertake well-established input-output mathematical calculations to estimate the direct impacts, as well as the indirect impacts throughout the infinite supply chains of a product, for example. As opposed to conventional life cycle assessments that are affected by so-called truncation errors because they only take into account selected supply chains, input-output analysis considers the entire supply chain of a product, technology or an organization in question, and this provides a complete picture of the impacts.

Qualitative sub-study (intervention group only)

We will conduct a qualitative evaluation after the 6 month follow up among a sub-sample of patients and clinicians. We will invite participants who were randomised to the intervention to indicate their interest in taking part in a interview (number of participants=30) to explore in depth their experiences with digitally supported self-examination. We will carry out face-to-face or telephone interviews among these patients at 6 months after randomisation. Interviews will be audio recorded and transcribed verbatim. We will also interview participating clinicians (n=5-10) to explore their experience of the benefits and limitations of the intervention. The study will take a Phenomenological perspective and will use Framework Analysis,[23] a matrix based method of thematic analysis which has been used successfully in numerous screening studies.[24,25]

13.6. Missing data

Missing data will be imputed in analysis as appropriate.

13.7. Deviations from original statistical plan

The version of this document and the statistical analyses plan will be updated with each change, and each change noted.

14. Responsibilities of the Principal Investigator at each site

The study will be performed in accordance with the CPMP/ICH Note for Guidance on Good Clinical Practice (CPMP/ICH/135/95) in Australia and applicable guidelines in participating sites overseas.

The study protocol, including the final version of the participant information and informed consent form to be used, must be approved by the Trial Management Committee and a constituted Human Research Ethics Committee (HREC) prior to enrolment of any patients into the study. The opinion of the HREC should be dated and given in writing. It is the responsibility of the PI at each site to forward a copy of the approval from the HREC clearly identifying the protocol submitted for review and a copy of the approved participant information sheet and consent form to the Trial Coordinating Centre prior to entry of patients.

The investigator is responsible for ensuring that written informed consent by the patient is obtained before study entry. The investigator is responsible for informing the Trial Coordinating centre and the HREC of any SAEs and/or major amendments to the protocol as per local requirements.

The investigator is responsible for ensuring that all regulatory requirements are followed.

The investigator is required to ensure compliance with the protocol in its entirety. It is the responsibility of the investigator to maintain adequate and accurate CRFs.

15. Reporting of Results

The Study Chair and TMC will be responsible for decisions regarding presentations and publications arising from this study.

Authorship credit should be based on the Vancouver statement by the International Committee of Medical Journal Editors, i.e. substantial contribution to all three of the following criteria:

- Conception and design OR analysis and interpretation
- Drafting article OR critically revising it for intellectual content
- Final approval of version to be published

Or, a fourth criterion is:

- Contributors who register 5% or more (accrual by institution) of the evaluable cases on a study will be listed as authors. The designated author is the choice of the institution's PI and in most cases would be the investigator with the highest accrual. If an institution places a large number of cases on the study, that institution will get an additional author for every 10% of the participants accrued, not to exceed a total of three authors (i.e. two authors for $\geq$ 15% accrual and three authors for $\geq$ 25% accrual)

Acknowledgement of the collaboration between MASC Trials is required in all publications, abstracts and presentations. Publications and abstracts must be presented to the Study Chair and TMC for review and approved prior to submission. Draft publications will be presented to the Publications/Writing Committee of each collaborating group for comment prior to submission.

Publications must be reviewed by the MASC Trials Executive Committee prior to submission.

16. Quality Assurance

16.1. Data Management and Source Data Verification

Trial sites are expected to regularly provide the completed CRFs reflecting the patient visits to the Trial Coordinating Centre. Copies of relevant documents for source verification and quality assurance will be requested including various imaging scans and reports.

The Trial Coordinating Centre will issue data queries as required to clarify CRF data and will report to the Study Chair regarding CRF submission and query completion rates as well as any issues related to protocol compliance.

16.2. Protocol Amendments

Changes and amendments to the protocol can only be made by the TMC. Approval of amendments by the HREC is required prior to their implementation. In some instances, an amendment may require a change to the participant information sheet and/or consent form. The Investigator must receive approval/advice of the revised consent form prior to implementation of the change. In addition, changes to the CRFs, if required, will be incorporated in the amendment.

16.3. On Site Monitoring

Site monitoring is scheduled annually for this study (also subject to funding and recruitment rate and at the discretion of the TMC).

16.4. Site Audits

This study is subject to audit by each of the groups involved and could occur at any stage of the study. Sites will be informed in advance in writing, outlining and the scope of the audit should one occur.

16.5. Confidentiality

The study will be conducted in accordance with applicable Privacy Acts and Regulations. All data generated in this study will remain confidential. All information will be stored and backed-up securely at the MASC Trials Coordinating Centre or at the School of Public Health at University of Sydney and will only be available to staff directly involved with the study. Hard-copy documents will be stored in locked cabinets. Electronic files will be stored on secure servers and files containing identifying information are password-protected.

Personal data identifying trial subjects will be held securely at the sites according to local institutional requirements for the purpose of follow up after the conclusion of the protocol-specified period. Sites may be asked to submit copies of source documents to the Trial Coordinating Centre e.g. pathology reports, however, all reports must be de-identified prior to sending, with only participant trial number and initials detailed.

17. References

1. Welfare AIoHa: Cancer incidence projections, Australia 2011 to 2020. Cancer series no. 66. . Canberra, 2012
2. Speijers MJ, Francken AB, Hoekstra-Weebers JE, et al: Optimal follow-up for melanoma. Expert Review of Dermatology 5:461-478, 2010
3. Australia Cancer Network & New Zealand Guidelines Group: Clinical Practice Guidelines for the Management of Melanoma in Australia and New Zealand, 2008
4. Coory M, Baade P, Aitken J, et al: Trends for in situ and invasive melanoma in Queensland, Australia, 1982-2002. Cancer Causes Control 17:21-7, 2006
5. Morton RL, Rychetnik L, McCaffery K, et al: Patients' perspectives of long-term follow-up for localised cutaneous melanoma. Eur J Surg Oncol 39:297-303, 2013

6. Rychetnik L, McCaffery K, Morton R, et al: Psychosocial aspects of post-treatment follow-up for stage I/II melanoma: a systematic review of the literature. Psychooncology 22:721-36, 2013

7. Watts CG, Cust AE, Menzies SW, et al: Specialized surveillance for individuals at high risk for melanoma: A cost analysis of a high-risk clinic. JAMA Dermatology, 2014

8. Turner RM, Bell KJL, Morton RL, et al: Optimizing the Frequency of Follow-Up Visits for Patients Treated for Localized Primary Cutaneous Melanoma. Journal of Clinical Oncology 29:4641-4646, 2011

9. Damude S, Hoekstra-Weebers JEHM, Francken AB, et al: The MELFO-Study: Prospective, Randomized, Clinical Trial for the Evaluation of a Stage-adjusted Reduced Follow-up Schedule in Cutaneous Melanoma Patients—Results after 1 Year. Annals of Surgical Oncology:1-10, 2016

10. Rychetnik L, McCaffery K, Morton RL, et al: Follow-up of early stage melanoma: specialist clinician perspectives on the functions of follow-up and implications for extending follow-up intervals. J Surg Oncol 107:463-8, 2013

11. Dummer R, Hauschild A, Guggenheim M, et al: Cutaneous melanoma: ESMO Clinical Practice Guidelines for diagnosis, treatment and follow-up. Annals of Oncology 23:vii86-vii91, 2012

12. Coups EJ, Manne SL, Stapleton JL, et al: Skin self-examination behaviors among individuals diagnosed with melanoma. Melanoma Res 26:71-6, 2016

13. Yagerman S, Marghoob A: Melanoma patient self-detection: a review of efficacy of the skin self-examination and patient-directed educational efforts. Expert Rev Anticancer Ther 13:1423-31, 2013

14. Simard S, Savard J: Fear of Cancer Recurrence Inventory: development and initial validation of a multidimensional measure of fear of cancer recurrence. Support Care Cancer 17:241-51, 2009

15. Murchie P, Allan JL, Brant W, et al: Total skin self-examination at home for people treated for cutaneous melanoma: development and pilot of a digital intervention. BMJ Open 5:e007993, 2015

16. Manahan MN, Soyer HP, Loescher LJ, et al: A pilot trial of mobile, patient-performed teledermoscopy. Br J Dermatol 172:1072-80, 2015

17. Lovibond SH, Lovibond, P.F. : Manual for the Depression Anxiety Stress Scales. (ed 2nd. Ed.). Sydney, Sydney Psychology Foundation, 1995

18. Dworkin SL: Sample size policy for qualitative studies using in-depth interviews. Arch Sex Behav 41:1319-20, 2012

19. Memari N, Hayen A, Bell KJ, et al: How Often Do Patients with Localized Melanoma Attend Follow-Up at a Specialist Center? Ann Surg Oncol 22 Suppl 3:S1164-71, 2015

20. Watts CG, Cust AE, Menzies SW, et al: Specialized surveillance for individuals at high risk for melanoma: a cost analysis of a high-risk clinic. JAMA Dermatol 151:178-86, 2015

21. Sanders GD, Neumann PJ, Basu A, et al: Recommendations for Conduct, Methodological Practices, and Reporting of Cost-effectiveness Analyses: Second Panel on Cost-Effectiveness in Health and Medicine. JAMA 316:1093-103, 2016

22. Husereau D, Drummond M, Petrou S, et al: Consolidated Health Economic Evaluation Reporting Standards (CHEERS) statement. BMJ 346:f1049, 2013

23. Ritchie J LJ: Qualitative Research Practice: A Guide for Social Science Students and Researchers. London, Sage, 2003

24. Smith SK, Dixon A, Trevena L, et al: Exploring patient involvement in healthcare decision making across different education and functional health literacy groups. Soc Sci Med 69:1805-12, 2009

25. Waller J, McCaffery K, Kitchener H, et al: Women's experiences of repeated HPV testing in the context of cervical cancer screening: a qualitative study. Psychooncology 16:196-204, 2007

18. Appendices

Table 1: Schedule of Assessment: Time and Events Table:

Follow on next page 29.

**Study Treatment:**	**Screening:**	**Time:**	Staff Identify participants:<br>• Stage 0/I/II melanoma who are attending regular melanoma follow-up<br>• 18 years or older<br>• Sufficient English language skills<br>• Are able to self-examine<br>• No known cognitive impairment<br>• Follow up appointment planned
Site staff identify participants			
**Invitation package:**			
Informed consent to study participation	√	Before follow up appointment	
Patient Information Sheet	√		
Participation and Eligibility card	√		
Study Invitation Letter	√		
Prepaid envelope to return Consent	√		
Baseline Questionnaire (hardcopy)	√		
**Eligible patient returns consent and participation card.**			
**Site staff performs patient randomisation.**			

	**Time from randomisation:**	**Control Arm**	**Intervention Arm**
**Welcome Package sent to participants. This include the following items:**	+/- 7 days		
Dermatoscope (By mail or face-to-face)			√
Rubber attachment-(smartphone-specific) (By mail or face-to-face)			√
Instructions for MoleScope App and Dermatoscope			√
Participant diary for next 6 months and 6 prepaid envelopes		√	√
"Your Guide to Early Melanoma Detection" educational booklet		√	√
Baseline letter- Control		√	
Baseline letter - Intervention			√
**Text/email reminders for Self-Skin Examination with ASICA survey code.**	Every 2 months		√
**6 month Follow-up package:**	6 months +/- 7 days		
6 month Follow-up Questionnaire (Hardcopy) and prepaid envelopes (for those opting for paper version)		√	√
6 month Letter – Control with REDCap code updated (Online)		√	
6 month Letter- Intervention with REDCap code updated (Online)			√

935
