# Supplementary material for: Acceptability of a Hypothetical Reduction in Routinely Scheduled Clinic Visits Among Patients With History of a Localized Melanoma (MEL-SELF): Pilot Randomized Clinical Trial
Source: JMIR Dermatol. 2023 Jun 26;6:e45865. doi: 10.2196/45865 (PMC10335154; doi:10.2196/45865)
Supplement: Multimedia Appendix 2 [file derma_v6i1e45865_app2.docx]

| **Appendix 2: Survey questions relating to hypothetical reduction in routinely scheduled clinic visits (delivered online via REDCap, Vanderbilt University [6,7])**  In this section we ask you to consider the hypothetical (or imaginary) scenario where you have fewer scheduled routine follow-up visits with your melanoma doctor(s). In other words, having scheduled skin clinic appointments less often. These questions assume that you may initiate unscheduled visits yourself as often as you need. Please read each statement and indicate how **acceptable** a **reduction in scheduled visits** is by circling the appropriate number. | | | | | |
| --- | --- | --- | --- | --- | --- |
|  | **Not**  **at all acceptable** | **Slightly** | **Some-what** | **Very** | **Completely acceptable** |
| - How acceptable would a decrease in scheduled visits with your melanoma specialist be? | 0 | 1 | 2 | 3 | 4 |
| - How acceptable would a decrease in scheduled visits with your GP be? | 0 | 1 | 2 | 3 | 4 |
| - How acceptable would a decrease in scheduled visits with all melanoma doctors (specialists and GP) be? | 0 | 1 | 2 | 3 | 4 |
| **Please provide reasons for why fewer scheduled routine follow up would be / would not be acceptable to you. In what circumstances would it be acceptable?** | | | | | |
