# Supplementary material for: Acceptability of a Hypothetical Reduction in Routinely Scheduled Clinic Visits Among Patients With History of a Localized Melanoma (MEL-SELF): Pilot Randomized Clinical Trial
Source: JMIR Dermatol. 2023 Jun 26;6:e45865. doi: 10.2196/45865 (PMC10335154; doi:10.2196/45865)
Supplement: Multimedia Appendix 3 [file derma_v6i1e45865_app3.docx]

**Appendix 3: Flow of participants in the MEL-SELF pilot randomised controlled trial**

Assessed for eligibility (n=481)

Assessed for outcome 1 (n=481)

Excluded (n=381)

♦ Inclusion criteria not met (n=116)

No smartphone (n=42)

Unable to perform SSE (n=35)

Not stage 0/I/II melanoma (n=18)

Not confident with technology (n=9)

Unknown / other (n=12)

♦ Declined to participate (n=47)

♦ Other (n=45)

♦ Unknown (n=173)

Assessed for outcome: Acceptability of reducing scheduled clinic visit frequency (n=36)

Lost to follow-up (n= 5)

Discontinued intervention (n=8)

♦Withdrawn (n=4)

♦Not enough time (n = 3)

♦Other (n=1)

Δ

Allocated to **control** (n=51)

♦ Received allocated intervention (n=49)

♦ Did not receive allocated intervention (n=2)

♦Inclusion criteria not met (n=2)

Lost to follow-up (n=5)

Discontinued intervention (n=11)

♦ Withdrawn (n=4)

♦ Not enough time (n=4)

♦ Other (n=3)

Allocated to **intervention** (n=49)

♦ Received allocated intervention (n=46)

♦ Did not receive allocated intervention (n= 3)

♦ Inclusion criteria not met (n=3)

Assessed for outcome: Acceptability of reducing scheduled clinic visit frequency (n=30)

## Allocation

## Assessment

## Follow-Up

Randomised (n=100)

## Screened

Assessed for eligibility (n=481)

Excluded (n=381)

♦ Inclusion criteria not met (n=116)

No smartphone (n=42)

Unable to perform SSE (n=35)

Not stage 0/I/II melanoma (n=18)

Not confident with technology (n=9)

Unknown / other (n=12)

♦ Declined to participate (n=47)

♦ Other (n=45)

♦ Unknown (n=173)

Assessed for objective 2 (n=36)

Assessed for objective 3 (n=36)

Assessed for objective 4 (n=33)

Assessed for objective 5 (n=35)

Assessed for objective 6 (n=36)

Assessed for objective 8 (n=51)

Assessed for objective 9 (n=51)

gg

, MF

Lost to follow-up (n= 5)

Discontinued intervention (n=8)

♦Withdrawn (n=4)

♦Not enough time (n = 3)

♦Other (n=1)

Δ

Allocated to **control** (n=51)

♦ Received allocated intervention (n=49)

♦ Did not receive allocated intervention (n=2)

♦Met exclusion criteria (n=2)

Lost to follow-up (n=5)

Discontinued intervention (n=11)

♦ Withdrawn (n=4)

♦ Not enough time (n=4)

♦ Other (n=3)

Allocated to **intervention** (n=49)

♦ Received allocated intervention (n=46)

♦ Did not receive allocated intervention (n= 3)

♦ Inclusion criteria not met (n=3)

Assessed for objective 2 (n=30)

Assessed for objective 3 (n=30)

Assessed for objective 4 (n=30)

Assessed for objective 5 (n=30)

Assessed for objective 6 (n=30)

Assessed for objective 7 (n=49)

Assessed for objective 8 (n=49)

Assessed for objective 9 (n= 49)

## Allocation

## Assessment

## Follow-Up

Randomised (n=100)

## Screened

Assessed for eligibility (n=481)

Excluded (n=381)

♦ Inclusion criteria not met (n=116)

No smartphone (n=42)

Unable to perform SSE (n=35)

Not stage 0/I/II melanoma (n=18)

Not confident with technology (n=9)

Unknown / other (n=12)

♦ Declined to participate (n=47)

♦ Other (n=45)

♦ Unknown (n=173)

Assessed for objective 2 (n=36)

Assessed for objective 3 (n=36)

Assessed for objective 4 (n=33)

Assessed for objective 5 (n=35)

Assessed for objective 6 (n=36)

Assessed for objective 8 (n=51)

Assessed for objective 9 (n=51)

gg

, MF

Lost to follow-up (n= 5)

Discontinued intervention (n=8)

♦Withdrawn (n=4)

♦Not enough time (n = 3)

♦Other (n=1)

Δ

Allocated to **control** (n=51)

♦ Received allocated intervention (n=49)

♦ Did not receive allocated intervention (n=2)

♦Met exclusion criteria (n=2)

Lost to follow-up (n=5)

Discontinued intervention (n=11)

♦ Withdrawn (n=4)

♦ Not enough time (n=4)

♦ Other (n=3)

Allocated to **intervention** (n=49)

♦ Received allocated intervention (n=46)

♦ Did not receive allocated intervention (n= 3)

♦ Inclusion criteria not met (n=3)

Assessed for objective 2 (n=30)

Assessed for objective 3 (n=30)

Assessed for objective 4 (n=30)

Assessed for objective 5 (n=30)

Assessed for objective 6 (n=30)

Assessed for objective 7 (n=49)

Assessed for objective 8 (n=49)

Assessed for objective 9 (n= 49)

## Allocation

## Assessment

## Follow-Up

Randomised (n=100)

## Screened

Assessed for eligibility (n=481)

Excluded (n=381)

♦ Inclusion criteria not met (n=116)

No smartphone (n=42)

Unable to perform SSE (n=35)

Not stage 0/I/II melanoma (n=18)

Not confident with technology (n=9)

Unknown / other (n=12)

♦ Declined to participate (n=47)

♦ Other (n=45)

♦ Unknown (n=173)

Assessed for objective 2 (n=36)

Assessed for objective 3 (n=36)

Assessed for objective 4 (n=33)

Assessed for objective 5 (n=35)

Assessed for objective 6 (n=36)

Assessed for objective 8 (n=51)

Assessed for objective 9 (n=51)

gg

, MF

Lost to follow-up (n= 5)

Discontinued intervention (n=8)

♦Withdrawn (n=4)

♦Not enough time (n = 3)

♦Other (n=1)

Δ

Allocated to **control** (n=51)

♦ Received allocated intervention (n=49)

♦ Did not receive allocated intervention (n=2)

♦Met exclusion criteria (n=2)

Lost to follow-up (n=5)

Discontinued intervention (n=11)

♦ Withdrawn (n=4)

♦ Not enough time (n=4)

♦ Other (n=3)

Allocated to **intervention** (n=49)

♦ Received allocated intervention (n=46)

♦ Did not receive allocated intervention (n= 3)

♦ Inclusion criteria not met (n=3)

Assessed for objective 2 (n=30)

Assessed for objective 3 (n=30)

Assessed for objective 4 (n=30)

Assessed for objective 5 (n=30)

Assessed for objective 6 (n=30)

Assessed for objective 7 (n=49)

Assessed for objective 8 (n=49)

Assessed for objective 9 (n= 49)

## Allocation

## Assessment

## Follow-Up

Randomised (n=100)

## Screened
