# Supplementary material for: Acceptability of a Hypothetical Reduction in Routinely Scheduled Clinic Visits Among Patients With History of a Localized Melanoma (MEL-SELF): Pilot Randomized Clinical Trial
Source: JMIR Dermatol. 2023 Jun 26;6:e45865. doi: 10.2196/45865 (PMC10335154; doi:10.2196/45865)
Supplement: Multimedia Appendix 4 [file derma_v6i1e45865_app4.docx]

*Appendix 4: Acceptability of a hypothetical decrease in routinely scheduled visits at 6 months follow up in participants with melanoma in situ vs participants with invasive melanoma^1,2^*

|  | **Melanoma in situ (n=36)** | | **Invasive Melanoma (n=64)^3^** | | **Total (n=100)** | |
| --- | --- | --- | --- | --- | --- | --- |
|  | **Baseline** | **Follow-up** | **Baseline** | **Follow-up** | **Baseline** | **Follow-up** |
| **Acceptability of a decrease in scheduled visits with all melanoma doctors (specialists and GP)** |  |  |  |  |  |  |
| Not acceptable | 17 (47%) | 14 (39%) | 27 (42%) | 18 (28%) | 44 (44%) | 32 (32%) |
| Slightly/somewhat acceptable | 13 (36%) | 10 (28%) | 22 (34%) | 19 (30%) | 35 (35%) | 29 (29%) |
| Very/completely acceptable | 1 (3%) | 1 (3%) | 7 (11%) | 4 (6%) | 8 (8%) | 5 (5%) |
| **Acceptability of a decrease in scheduled visits with GP** |  |  |  |  |  |  |
| Not acceptable | 11 (31%) | 9 (25%) | 13 (20%) | 9 (14%) | 24 (24%) | 18 (18%) |
| Slightly/somewhat acceptable | 12 (33%) | 14 (39%) | 26 (41%) | 20 (31%) | 38 (38%) | 34 (34%) |
| Very/completely acceptable | 8 (22%) | 2 (6%) | 17 (27%) | 12 (19%) | 25 (25%) | 14 (14%) |
| **Acceptability of a decrease in scheduled visits with melanoma specialist** |  |  |  |  |  |  |
| Not acceptable | 16 (44%) | 14 (39%) | 27 (42%) | 19 (30%) | 42 (42%) | 33 (33%) |
| Slightly/somewhat acceptable | 14 (39%) | 10 (28%) | 20 (31%) | 17 (27%) | 34 (34%) | 27 (27%) |
| Very/completely acceptable | 1 (3%) | 1 (3%) | 9 (14%) | 5 (8%) | 10 (10%) | 6 (6%) |
| ^1^ Percentages may not sum to 100 owing to rounding.  ^2^ Missing data at baseline for 5 (14%) participants in the melanoma in situ group and 8 (13%) participants in the invasive melanoma group; and at follow-up for 11 (31%) in the melanoma in situ group and 23 (36%) in the invasive melanoma group.  ^3^ Invasive melanoma includes participants with American Joint Committee on Cancer (AJCC) stages I, II, or III/IV and 1 participant whose AJCC stage was unknown. | | | | | | |
